# Impact of Biohybrid Magnetite Nanoparticles and Moroccan Propolis on Adherence of Methicillin Resistant Strains of *Staphylococcus aureus*

**DOI:** 10.3390/molecules21091208

**Published:** 2016-09-09

**Authors:** Soukaina El-Guendouz, Smail Aazza, Badiaa Lyoussi, Vassya Bankova, João P. Lourenço, Ana M. Rosa Costa, José F. Mariano, Maria G. Miguel, Maria L. Faleiro

**Affiliations:** 1 Laboratory of Physiology-Pharmacology-Environmental Health, Faculty of Sciences Dhar El Mehraz, BP 1796 Atlas, University Sidi Mohamed Ben Abdallah, Fez 30 000, Morocco; soukaina.elguendouz@gmail.com (S.E.G.); aazzasmail@ymail.com (S.A.); lyoussi@gmail.com (B.L.); 2 Department of Chemistry and Pharmacy, Faculty of Science and Technology, University of Algarve, Campus de Gambelas, MeditBio, Faro 8005-139, Portugal; 3 Institute of Organic Chemistry with Centre of Phytochemistry, Acad. G. Bonchev strl. bl. 9, Sofia 1113, Bulgaria; bankova@orgchm.bas.bg; 4 Algarve Chemistry Research Centre(CIQA) and Department of Chemistry and Pharmacy, Faculty of Science and Technology, University of Algarve, Faro 8005-139, Portugal; jlouren@ualg.pt (J.P.L.); amcosta@ualg.pt (A.M.R.C.); 5 CQE, Centro de Química Estrutural, Instituto Superior Técnico, University of Lisbon, Av. Rovisco Pais, Lisboa 1049-001, Portugal; 6 Department of Physics and CeFEMA, Faculty of Science and Technology, University of Algarve, Campus de Gambelas, Faro 8005-139, Portugal; jmariano@ualg.pt; 7 Department of Biological Sciences and Bioengineering, Faculty of Science and Technology, University of Algarve, Center for Biomedical Research, Faro 8005-139, Portugal; mfaleiro@ualg.pt

**Keywords:** co-precipitation, hydrothermal, functionalization, propolis, flavonoids, diterpenes

## Abstract

Biofilm bacteria are more resistant to antibiotics than planktonic cells. Propolis possesses antimicrobial activity. Generally, nanoparticles containing heavy metals possess antimicrobial and antibiofilm properties. In this study, the ability of adherence of Methicillin Resistant Strains of *Staphylococcus aureus* (MRSA) to catheters treated with magnetite nanoparticles (MNPs), produced by three methods and functionalized with oleic acid and a hydro-alcoholic extract of propolis from Morocco, was evaluated. The chemical composition of propolis was established by gas chromatography mass spectrometry (GC-MS), and the fabricated nanostructures characterized by X-ray diffraction (XRD), transmission electron microscopy (TEM), Mossbauer spectroscopy and Fourrier transform infrared spectroscopy (FTIR). The capacity for impairing biofilm formation was dependent on the strain, as well as on the mode of production of MNPs. The co-precipitation method of MNPs fabrication using Fe^3+^ and Na_2_SO_3_ solution and functionalized with oleic acid and propolis was the most effective in the impairment of adherence of all MRSA strains to catheters (*p* < 0.001). The adherence of the strain MRSA16 was also significantly lower (*p* < 0.001) when the catheters were treated with the hybrid MNPs with oleic acid produced by a hydrothermal method. The anti-MRSA observed can be attributed to the presence of benzyl caffeate, pinocembrin, galangin, and isocupressic acid in propolis extract, along with MNPs. However, for MRSA16, the impairment of its adherence on catheters may only be attributed to the hybrid MNPs with oleic acid, since very small amount, if any at all of propolis compounds were added to the MNPs.

## 1. Introduction

Propolis is a natural resinous substance collected by honeybees from parts of plants, buds, and exudates, mixed with bees wax and salivary enzymes. Propolis is mainly constituted by resin (50%), wax (30%), essential oils (10%), pollen (5%), and other substances (5%), such as debris, minerals and organic compounds, despite the chemical variability that can be found. Such differences can be attributed to the different plants from where bees can collect the materials, as well as the different harvesting seasons of propolis. Polyphenols, terpenoids including steroids, naphthalene and stilbene derivatives, and fatty acids are some examples of organic compounds that can be found in propolis [1,2]. Different biological properties have been attributed to propolis, such as antimicrobial, anti-inflammatory, antioxidant, antidiabetic, spasmolytic, anaesthetic, anticancer, and immunomodulatory effects [2,3].

*Staphylococcus aureus* is a Gram-positive bacterium that mainly colonizes the nares and is considered a human pathogen that shows increasing incidence in patients with prolonged hospitalization, submitted to intravenous drugs or under treatment with enteral feedings or dialysis, in postoperative surgical wounds in decubitus ulcers or in indwelling catheters [4,5,6]. Its incidence is increasing also in the community [7]. Over time, *Staphylococcus aureus* has developed resistance to several antibiotics and, presently, methicillin resistant strains (MRSA) are causing serious infections in hospitals or even in the community [8,9]. For these reasons, alternative approaches are required to fight this bacterial pathogen, and one of them is the use of natural products. Propolis comes as a natural candidate due to its multidirectional mechanism of action on bacterial cells, which renders the development of resistance unlikely [10]. The utilization of propolis extracts has already been assayed as anti-MRSA agents, as is shown in Table 1, which compiles the results of several authors who observed anti-MRSA activity in propolis of diverse origins.

Biofilm bacteria (adherent bacterial cells) are more resistant to antibiotics, cleaning and eradication than planktonic cells (cells in suspension). The percentage of infections associated with biofilm formation in medical devices is more than 25%. These infections cause great mortality and morbidity rates in patients with indwelling or implanted devices, with the consequent increasing of costs due to the long term treatments [11].

Different types of nanoparticles have been reported as possessing antimicrobial and antibiofilm properties, particularly those containing heavy metals [12]. Iron oxide magnetic nanoparticles were approved by FDA for biomedical uses, because they occur naturally in liver, spleen and human heart, consequently, they will be non-toxic and biocompatible at physiological concentrations. In addition, the production of these nanoparticles is not difficult and they are chemically stable under physiological conditions. They are also easily functionalized. Functionalized iron oxide magnetic nanoparticles, such as magnetite nanoparticles (MNPs) have been evaluated for inhibiting microbial growth and biofilm formation of yeasts (*Candida albicans*, *C. tropicalis*, *C. krusei*, *C. glabrata*, and *Saccharomyces cerevisiae*), and bacteria (*Escherichia coli*, *Staphylococcus aureus*, *Pseudomonas aeruginosa*, and *Enterococcus faecalis*) [13].

The majority of the methods used for synthesizing stable, biocompatible and monodispersed MNPs include co-precipitation, thermal decomposition, hydrothermal synthesis, microemulsion, and sonochemical synthesis. However, other methods have been developed and reported: electrochemical synthesis, laser pyrolysis techniques, and microorganism or bacterial synthesis [14]. In order to prevent that MNPs form aggregates, and for raising its biocompatibility, stability and biological application, such NPs may be functionalized with some biomolecules [14]. Oleic acid has been used alone or along with natural products for preventing biofilm formation of pathogenic microorganisms (*C. albicans*, *C. tropicalis*, *S. aureus*, and *E. coli*) or for promoting its disruption [12,15,16].

The main goal of the present work was to evaluate the ability of a hybrid nanosystem constituted by magnetite nanoparticles, produced by three methods based on two methods (co-precipitation and hydrothermal synthesis) and functionalized with oleic acid and a hydro-alcoholic extract of propolis from Morocco to prevent the adherence of MRSA strains on catheters, and therefore impair the formation of biofilm.

## 2. Results

### 2.1. Chemical Composition of Propolis Extract

Table 2 depicts the chemical composition of the hydro-alcoholic extract of propolis. Flavonoids (31.9%), diterpenes (21.5%), and phenolic acid esters (16.5%) were the main groups of compounds found in this extract. Only four components were present at concentrations higher than 5%: the diterpene isocupressic acid (8.1%) (**1**), and the three flavonoids pinocembrin (7.4%) (**2**), pinocembrin chalcone (5.9%) (**3**), and galangin (5.3%) (**4**). The phenolic acid ester benzyl caffeate (4.7%) (**5**) was present in the extract at a concentration very close to 5% (Table 2).

### 2.2. Characterization of the Nanomaterial

#### 2.2.1. X-ray diffraction (XRD)

**Figure 1:**
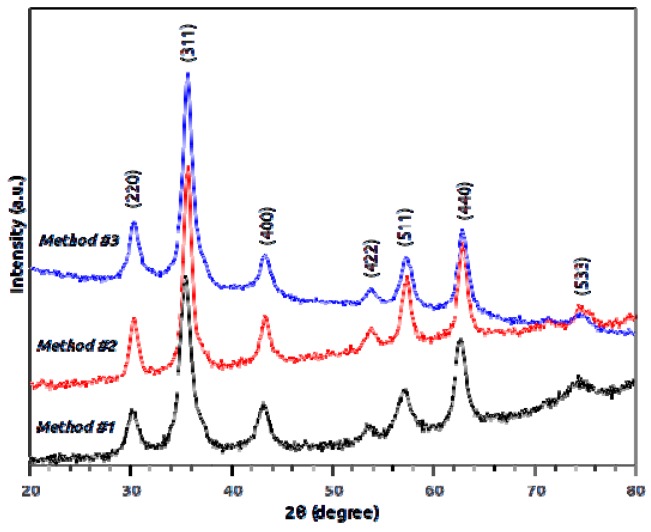
X-ray diffractograms of magnetite nanoparticles synthesized by the three methods. The labeled crystallographic planes refer to magnetite.

**Figure 2:**
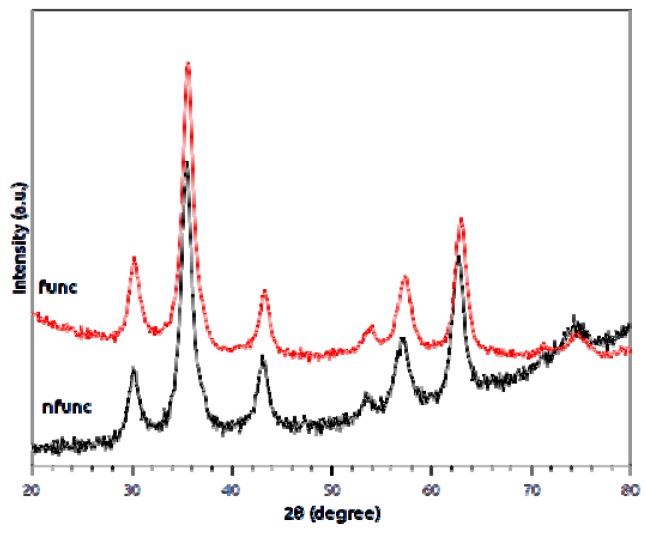
X-ray diffractograms of nonfunctionalized (nfunc) and functionalized with oleic acid (funct) magnetite nanoparticles, synthesized using Method #1.

Figure 1 displays the XRD patterns of the non-functionalized nanoparticles synthesized by each of the three methods. The diffraction peaks present in the patterns of all the samples indicate magnetite as the sole crystalline phase. The smaller width of the peaks in the pattern of Method #2 suggests a larger size of the nanoparticles in that sample, in agreement with transmission electron microscopy (TEM) observations. Figure 2 represents the diffractograms of both functionalized and nonfunctionalized nanoparticles, synthesized by Method #1, where it is evident that the functionalization procedure does not change the structure and composition of the nanoparticles.

#### 2.2.2. TEM

**Figure 3:**
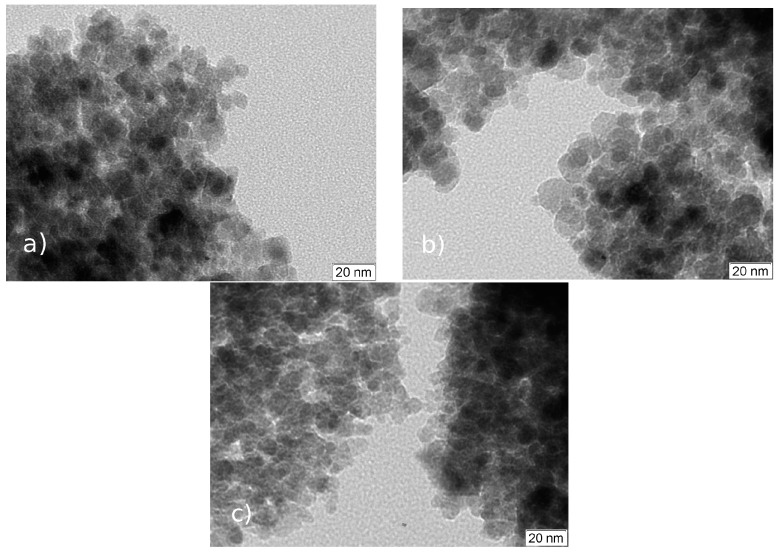
TEM micrographs of the nonfunctionalized nanoparticles synthezised by Methods: #1 (**a**); #2 (**b**); and #3 (**c**).

This analysis was used to characterize the morphology of the nonfunctionalized nanoparticles and the results are presented in Figure 3. The images show that the three methods produced roughly spherical nanoparticles, although those produced by Method #2 are of more regular shape, with diameters of about 15 nm, while Method #1 originates more angular shaped nanoparticles, with estimated sizes of 10 nm and Method #3 produces nanoparticles with approximately the same size but with a more regular spherical shape.

#### 2.2.3. Mössbauer Spectroscopy

**Figure 4:**
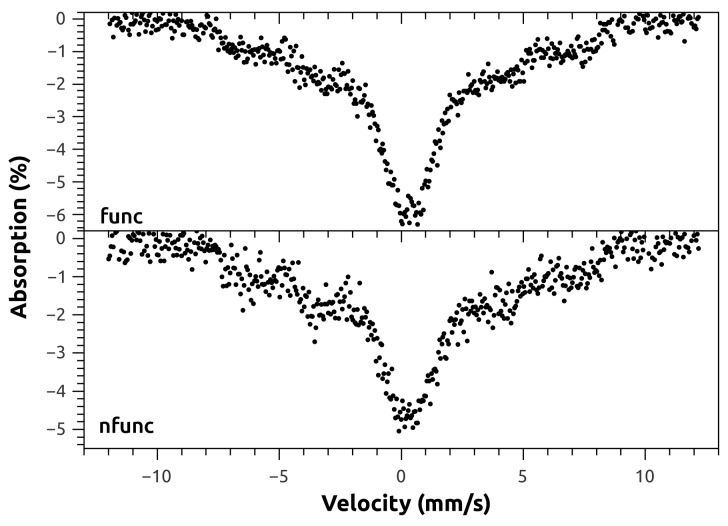
Room temperature ^57^Fe Mössbauer spectra of magnetite nanoparticles, nonfunctionalized and functionalized with oleic acid, synthesized by Method #1. The acquisition time is different for the two spectra.

In order to investigate the possible alteration of structure and composition by the functionalization procedure, two samples of magnetite synthesized by Method #1, with and without functionalization with oleic acid, were subject to Mössbauer analysis. Figure 4 shows the ^57^Fe Mössbauer spectra of these samples. As can be seen, no apparent alteration on the spectra was introduced by the functionalization of the nanoparticles, which agrees with the XRD results.

**Figure 5:**
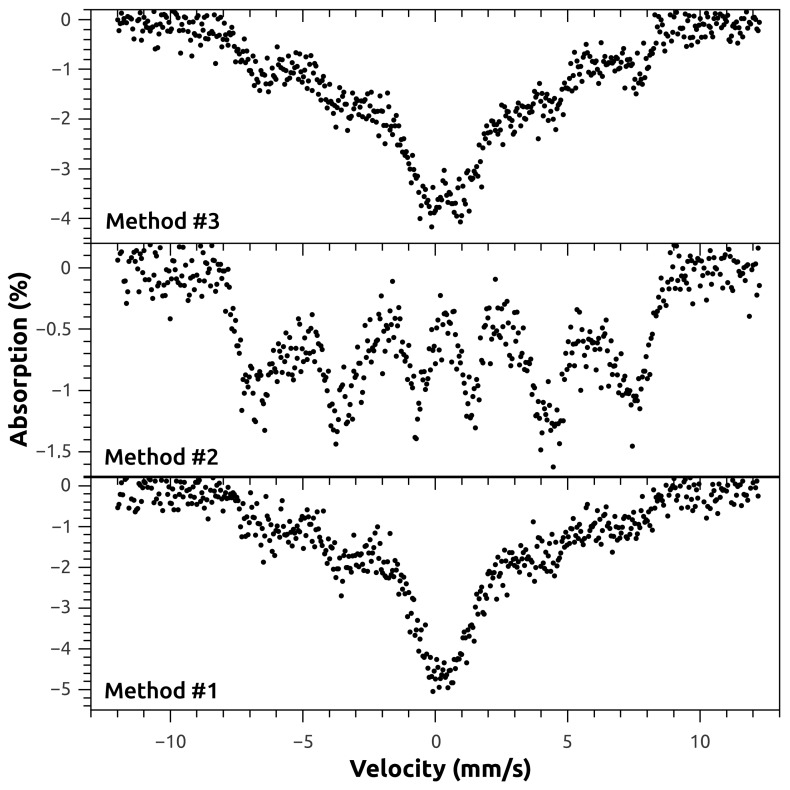
Room temperature ^57^Fe Mössbauer spectra of the nonfunctionalized iron oxide nanoparticles synthesized by the tree different methods. The spectra were taken with different acquisition times.

To further investigate the structure and composition of the as-synthesized MNPs, one sample of uncoated MNP synthesized by each of the three methods was analyzed by transmission Mössbauer spectroscopy. The recorded spectra are shown in Figure 5. The room temperature spectra show the gradual passage from bulk-like to superparamagnetic (SPM) behavior, as the average size of the particles decrease. The Method #2 spectra displays a single sextet compatible with magnetite, with board lines indicating that these particles have a multidomain structure, although close to the single domain size limit. In the Method #3 sample, the central doublet evidences a predominant SPM size, with some contribution from the non-SPM particles sextet, correlating to a size distribution with smaller average particle size. Sample from Method #1 exhibits a similar behavior. Thus, it can be stated from the Mössbauer analysis that Method #2 produces Fe_3_O_4_ NPs that have on average a larger size than those produced by Methods #1 and #3, which is in good agreement with the results of both TEM and XRD.

#### 2.2.4. Fourrier Tranform Infrared Spectroscopy (FTIR)

**Figure 6:**
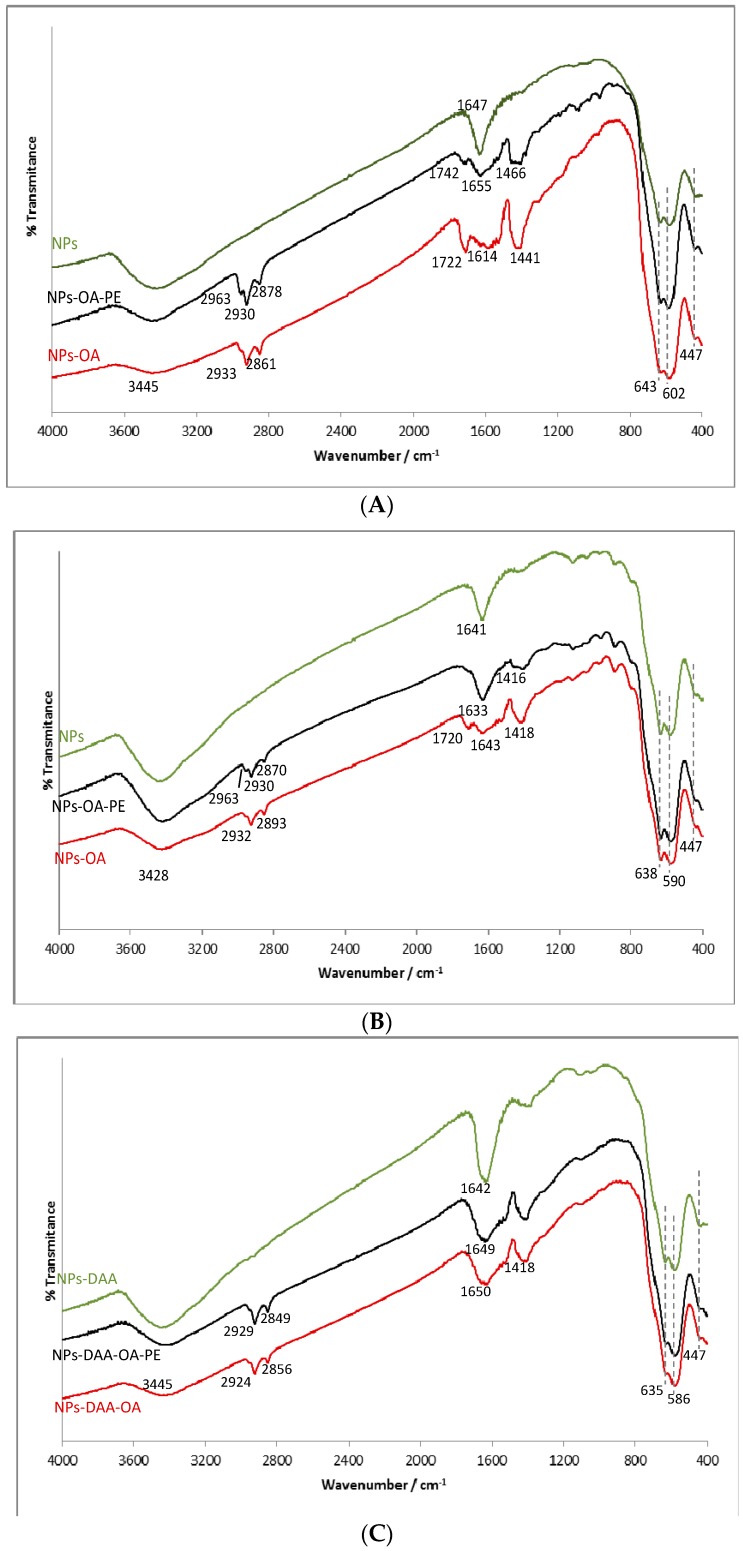
FTIR spectra of as obtained and functionalized magnetite NPs by: (**A**) Method #1; (**B**) Method $2; and (**C**) Method $3. OA—oleic acid, PE—propolis extract, DAA—dehydroascorbic acid.

In the FTIR spectra of MNPs prepared by Methods #1 and #2 (Figure 6A,B), an intense band near 600 cm^−1^ and the one centered at 447 cm^−1^ were observed. A splitting of the former into two bands (approximately 590 and 640 cm^−1^) was observed. Besides these bands, two other are observed in these spectra: one centered near 3445 cm^−1^ and the other around 1640 cm^−1^.

The FTIR spectrum of MNPs prepared by Method #3 (Figure 6C) does not differ much from the former, as the only significant absorption band of dehydroascorbic acid, the oxidation product of ascorbic acid, is from the carbonyl groups, at 1680 cm^−1^. As a consequence, only a broadening of the 1640 cm^−1^ band relative to the other NPs samples is observed. Apart from that, two inconspicuous bands, due to C-H asymmetric and symmetric stretching, may be denoted at nearly 2930 and 2850 cm^−1^, respectively.

Upon functionalization with oleic acid, the spectra of all MNPs show changes, indicating the presence of the fatty acid: the appearance of the C-H asymmetric and symmetric stretching bands, near 2930 and 2870 cm^−1^, a broadening of the band at 1640 cm^−1^, and a new band centered around 1430 cm^−1^, due to the bending of the methylene groups in OA chain. The broadening of the 1640 cm^−1^ band is due to a shift in the C=O stretching band, originally near 1710 cm^−1^, to lower wavenumbers, upon binding to the surface, that weakens the double bond character of the carbonyl. Nevertheless, in some samples (MNPs obtained by Methods #1 and #2), a band at 1720 cm^−1^ was still visible. After addition of propolis extract, this band remains in the first sample, although less intense, but disappears or at least fades away in the second one. In MNPs prepared by Method #3 this band is not present, meaning that the presence of dehydroascorbic acid somehow prevented the formation of an oleic acid bilayer, in the same manner as the compounds present in the propolis extract, that seem to decrease the amount of unbound oleic acid. 

The effect of the addition of propolis extract on the FTIR spectra is mainly related to the aromatic compounds, since the aliphatic ones, acids, and esters absorb in the same regions as the compounds already present. Therefore, only a new band at 2963 cm^−1^, attributable to the C-H stretching of unsaturated and aromatic hydrocarbons, as well as a broadening in the band centered near 1430 cm^−1^, due to the C=C stretching absorption (in the region 1450–1470 cm^−1^) are noteworthy in the spectra of the two first samples. In the last one, the spectra before and after addition of propolis extract seem practically superimposable, which means that only a very small amount, if any at all, of propolis compounds were added to the MNPs.

### 2.3. Impact of the Nanoparticles on Bacterial Adherence

**Figure 7:**
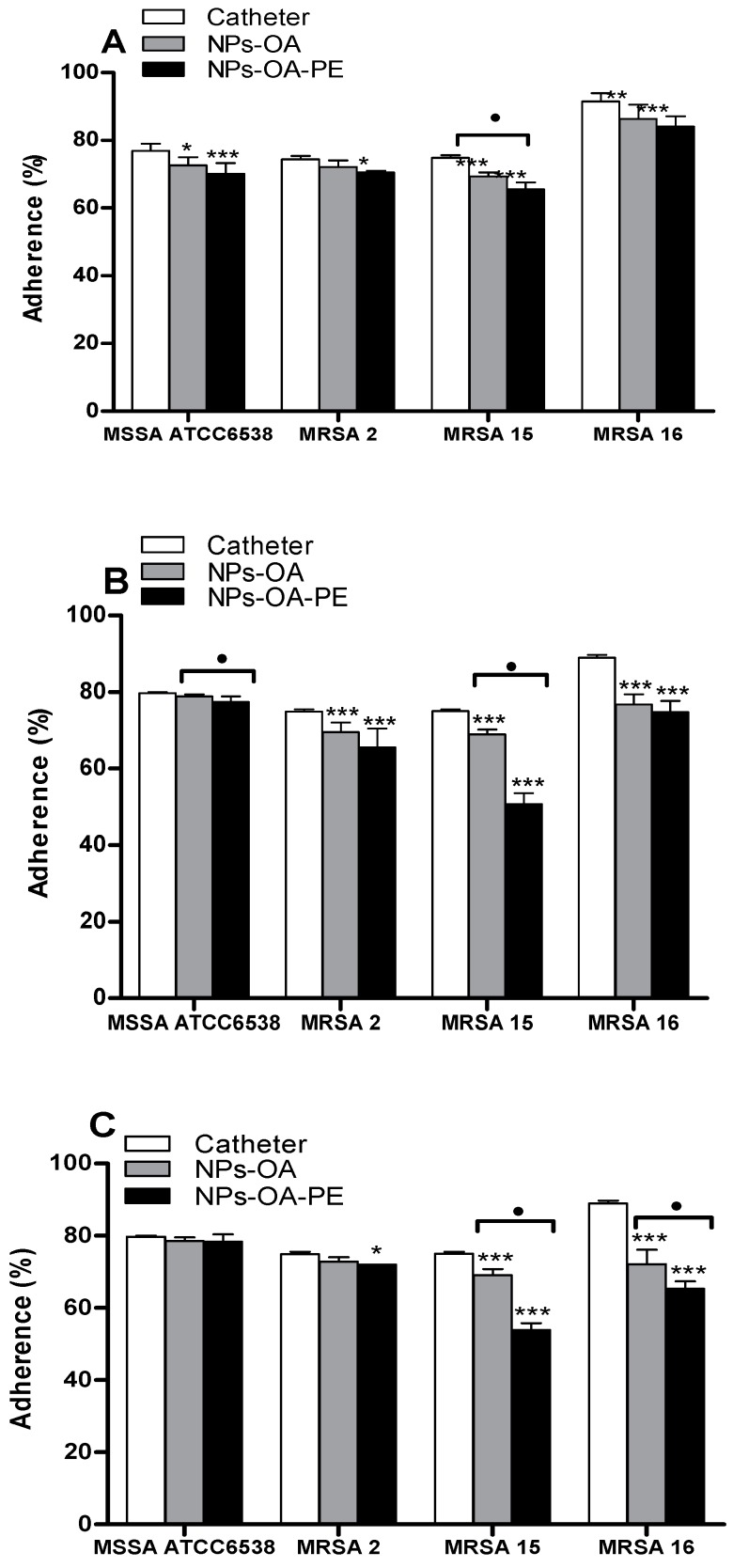
Percentage of adherence of microorganisms on catheter after contact with functionalized MNPs obtained by different methods: (**A**) Method #1; (**B**) Method #2; and (**C**) Method $3. OA—oleic acid, PE—propolis extract, Data represent the mean ± S.D from two separated experiments, * *p* < 0.05; ** *p* < 0.01; *** *p* < 0.001 (*n* = 6), statistically significant when compared with catheter not submitted to any treatment. Square brackets indicate the use of One Way ANOVA for each group. ^●^ *p* < 0.05 (*n* = 6).

The effect of the nanoparticles on bacterial adherence was evaluated using catheters treated with different MNPs. The results are illustrated in Figure 7A–C. The percentage of adherence of the cells of the MSSA strain was not affected by Methods #2 and #3 of nanoparticles production in comparison to the MRSA strains. However, the adherence of this strain was significantly lower (*p* < 0.05) by Method #1, particularly in the presence of propolis (*p* < 0.001).

The impact of the nanoparticles against the adherence ability of MRSA strains was dependent of the method of production of the nanoparticles and the strain, being the Method #2 the more efficient in inhibiting adherence of MRSA strains, particularly MRSA 15, which adherence was significantly lower (*p* < 0.001) when the catheters were treated with MNPs functionalized with oleic acid and propolis extract (50.67% ± 2.90%). The adherence of MRSA 2 was also impaired by the nanoparticles produced by the Method #2 (*p* < 0.001). The adherence of the strain MRSA 16 was significantly lower (*p* < 0.001) when the catheters were treated with magnetite nanoparticles produced by Method #3 either just with oleic acid or nanoparticles with oleic acid and propolis extract.

Thus, in order to impair the adherence of MRSA strains, the use MNPs produced by the Method #2 and functionalized with oleic acid or oleic acid plus propolis extract are the more appropriate followed by the Method #3. To our best knowledge, this is the first study that shows that catheters treated with magnetite nanoparticles functionalized with oleic acid and Moroccan propolis can impair the adherence of such problematic pathogenic bacteria, as MRSA strains.

## 3. Discussion

The presence of diterpenes is common in propolis of Mediterranean origin [24]. This group of compounds was already reported in some Moroccan propolis [25]. Popova et al. [25] found at least three main types of propolis of Moroccan origin: flavonoid/phenolic acid esters-type, flavonoid-type, and diterpene-type. In the present work, flavonoid/diterpene-type, along with a relative high amount of phenolic acid esters, was found.

The characterization of the nanomaterials was followed by XRD, TEM, Mössbauer spectroscopy, and FTIR. The diffraction peaks present in the patterns of all the samples indicate magnetite as the sole crystalline phase [26] (ICDD PDF-2 card #01-071-6337). As reported by Mahdavi et al. [27] for functionalized MNPs with oleic acid, XRD data showed negligible effects of this fatty acid on the XRD spectra data. The morphology and particle size of nanoparticles were similar to those reported by Anghel et al. [15] for functionalized magnetite (Fe_3_O_4_/C_18_), that is, the same type of functionalization (oleic acid) done in the present work. In the Mössbauer spectroscopy, and at room temperature, spectra showed a gradual passage from bulk-like to superparamagnetic (SPM) behavior, as the average size of the particles decrease, a striking characteristic of the superparamagnetic nature of nanoparticles [28].

FTIR spectroscopy contributes to provide a fast way of identification of MNPs. This technology is also useful when there is their functionalization, because it allows verifying the occurrence of such functionalization. In the identification of the nanoparticles, and for the Methods #1 and #2 there was confirmation of the presence of MNPs due to the intense band near 600 cm^−1^, attributed to the stretching of the Fe-O bond of octahedral positions, and the one centered at 447 cm^−1^, due to the stretching of tetrahedral Fe-O [29]. The splitting of the intense band near 600 cm^−1^ in two bands (approximately 590 and 640 cm^−1^) is an effect of the finite size that causes the split of the vibrational energy levels in the nanoparticles [30]. Two other bands were observed: one centered near 3445 cm^−1^, attributed to the O-H stretching both in hydroxyl groups on the MNPs surface and in adsorbed water molecules, and the other around 1640 cm^−1^ is attributed to the bending in these molecules [30,31]. Upon functionalization with oleic acid, the spectra of all MNPs show changes, indicating the presence of the fatty acid, nevertheless, in some samples (MNPs obtained by Methods #1 and #2), a band at 1720 cm^−1^ was still visible, probably due to the presence of unbound oleic acid molecules, forming a second layer [30]. The effect of the addition of propolis extract on the FTIR spectra was mainly related to the aromatic compounds.

The dependence of the method of production of MNPs as well as the strain on the impact of the adherence ability of microorganisms observed in the present work was also reported by some authors [16,32], using other strains and methods of production of NPs. The different response of diverse strains to the same compound or extract is expectable since there are not universal antimicrobials. However in the presence of functionalized nanoparticles with the same compound or extract, care must be taken in the production of such NPs, because unexpected activities may occur.

Recently, some works have demonstrated the capacity of propolis extract to prevent the adherence of several microorganisms (*Streptococcus mutans*, and several *Staphylococcus aureus* clinical strains) on abiotic (smooth glass or microplate) and biotic (HEp-2 (Human Epithelioma) and HeLa (cervical carcinoma)) surfaces [33,34,35]. With the exception of Veloz et al. [34], in the remaining works, the authors did not describe the chemical composition of propolis extracts; only refer the importance of phenol content on the inhibition of biofilm formation [33], or there is not ever any reference to the composition of the propolis sample [35].

The anti-MRSA activity of propolis extracts from different origins and consequently with distinct chemical compositions has been reported, as can be seen in Table 1, nevertheless the anti-MRSA biofilm formation by propolis is very scarcely reported or even absent, as far as we know. The capacity for inhibiting the growth of MRSA by propolis extracts has been attributed to several compounds, including phenolic esters (benzyl caffeate) and flavonoids (pinocembrin, galangin and their derivatives), also detected in our sample, nevertheless it also presents isocupressic acid, a diterpene, in relative high amounts; which along with the other phenols may be responsible for the anti-adherence activity found.

It is noteworthy to refer some discrepancies on the anti-MRSA activities of some components present in propolis and found in different works developed by diverse authors. As examples: Boisard et al. [17] found that the flavonoids pinobanksin-3-acetate, pinocembrin, chrysin, and galangin did not show anti-MRSA activity, in contrast to the prenyl caffeate. Nevertheless, the activities of the extracts were higher than the isolated compounds, which evidence a synergetic activity among the compounds present in the extracts. These results disagree with those found by Darwish et al. [20] and Pepeljnjak and Kosalec [23]. These authors reported higher activity of pinobanksin-3-acetate, pinocembrin and galangin than crude extracts.

Functionalized Fe_3_O_4_/C_18_ nanoparticles without propolis extract present ability for inhibiting the adherence of MRSA strains, as already reported by Anghel et al. [15], nevertheless always inferior when compared to those samples also functionalized with propolis extract, which reveals the importance of this extract on the activity.

## 4. Materials and Methods

### 4.1. Chemicals and Reagents

Hydrochloric acid (HCl) and l-(+)-Ascorbic acid were purchased from Merck (Darmstadt, Germany). Na_2_CO_3_ and Na_2_SO_3_ were purchased from Riedel de Haen (Seelze, Germany). Chloroform (CHCl_3_) was purchased from LAB-SCAN (Dublin, Ireland). Oleic acid and Iron(II) sulfate heptahydrate (FeSO_4_·7H_2_O) were purchased from Sigma Aldrich (Steinheim, Germany). Iron(III) chloride anhydrous was purchased from Fluka Chemicals (Buchs, Switzerland). NaOH was from Pronalab (Madalena, Portugal). Ethanol was from Panreac Quimica (Barcelona, Spain). The medium brain-heart infusion (BHI) and Bacteriological agar type E were purchased from Biokar Diagnostics (Beauvais, France).

### 4.2. Propolis Extract

One gram of propolis from Morocco (region of Fez-Boulmane) was chopped into small pieces and extracted by maceration using 30 mL of 70% ethanol and maintained for one week at 37 °C under agitation (200 rpm). The resulting solution was filtered under vacuum. A clear solution, without further purification, was used for successive analyses.

### 4.3. GC-MS Analysis of Propolis Extract

The analysis was performed with a Hewlett-Packard gas chromatograph 5890 series II Plus linked to a Hewlett-Packard 5972 mass spectrometer (Hewlett-Packard, Wilmington, DE, USA) system equipped with a 30 m long, 0.25 mm i.d., and 0.5 µm film thickness HP5-MS capillary column. The work conditions were the same as previously reported [25]. Semi-quantification was carried out by internal normalization with the area of each compound. The addition of individual areas of the compounds corresponds to 100% area. Compound identification was performed using commercial libraries and comparison of mass spectra and retention times of reference compounds.

### 4.4. Preparation of Magnetite Nanoparticles

Magnetite nanoparticles were synthesized by three different methods.

#### 4.4.1. Method #1

The alkaline co-precipitation of iron salts in aqueous solutions, as described by Kang et al. [36], with some modifications, was as follows: 0.43 mL of HCl 12 M was diluted with 12.5 mL of distilled water, and then 2.6 g of FeCl_3_ and 2.4 g of FeSO_4_·7H_2_O (2:1 molar ratio) were both added under continuous magnetic stirring. The resulting solution was poured into 125 mL of NaOH (1.5 M) under vigorous stirring, which was maintained for 30 min. The last step generated an instant black precipitate. The magnetism was checked with a NdFeB permanent magnet. The precipitate was attracted by the magnetic field, and the supernatant was removed by decantation. The recovered magnetite was washed with distilled water for three times and then twice with ethanol. The product was air-dried overnight and grinded into powder.

#### 4.4.2. Method #2

The co-precipitation, according to the procedure of Qu et al. [37] slightly modified, was as follows: 30 mL of a 2 M FeCl_3_ stock solution, prepared by dissolving the necessary amount of iron salt in 2 M HCl, were diluted with an equal volume of deionized water. Afterwards, 20 mL of a 1 M Na_2_SO_3_ solution were added under stirring. Just after the mixing of Fe^3+^ and SO_3_^2−^, the color of the solution altered from light yellow to red, indicating the formation of a complex ion. Meanwhile, an ammonia solution was prepared by the dilution of 50.8 mL of concentrated ammonia to a final volume of 800 mL. The former solution was quickly poured into the diluted ammonia solution, under vigorous stirring, as soon as its color changed back from red to yellow again, as the complex decomposed to Fe^2+^ and SO_4_^2−^. A black precipitate formed immediately, but stirring was continued for 30 min. A permanent magnet was applied to the beaker containing the suspension, and a black powder could be seen to quickly settle on the bottom. The supernatant was discarded and fresh water was added to the beaker. After decantation, the powder was washed one time with an acidic solution (pH 3–4), then with distilled water until neutral pH, once with ethanol, and finally left to air dry overnight*.*

#### 4.4.3. Method #3

Water-soluble magnetite nanoparticles were obtained by a hydrothermal method, according to Xuan et al. [38], using a ferric salt as single iron precursor, and ascorbic acid as reducing agent. The so synthesized super paramagnetic Fe_3_O_4_ nanocrystals are capped with dehydroascorbic acid (the oxidized form of ascorbic acid). In a typical experiment, 0.33 g of FeCl_3_ were dissolved in 25 mL of H_2_O, under continuous stirring. Then, 10 mL of 0.6 M Na_2_CO_3_ (0.6 M) were added, drop by drop and, 10 min later, 0.12 g ascorbic acid were also added. After being stirred for another 15 min, the solution was transferred to a 40 mL Teflon sealed autoclave. The autoclave was kept at 160 °C for 3 h and then let cool down to room temperature. The final product was separated from the reaction medium by using a NdFeB permanent magnet. A rinsing process including three cycles of decantation/washing/decantation in deionized water and in alcohol was performed before air drying overnight.

### 4.5. Preparation of Functionalized Magnetite Nanoparticles

Each magnetite type was functionalized with 6 mL of oleic acid (OA), the mixture being heated up to 80 °C on a water bath, under intense stirring for 60 min. Excess oleic acid was phase separated by dropwise addition of 4 mL of water, followed by OA-coated magnetite washing twice with ethanol and kept to dry overnight [39].

### 4.6. Synthesis of A Hybrid Core/Shell/Coated Shell Nanomaterial

Functionalized magnetite was added into 10 mL of CHCl_3_ (0.33% *w*/*v*) and was sonicated for 30 min. One mL of propolis sample at the MIC value (0.36 mg/mL) was added into the same volume of the magnetite suspension.

### 4.7. Characterization of the Nanomaterial

Characterization of the nanomaterial was carried out by X-ray powder diffraction (XRD), transmission electron microscopy (TEM) (Hitachi High-Technologies Corporation, Tokyo, Japan), ^57^Fe Mossbauer spectroscopy, and Fourier Transform Infrared (FTIR) spectroscopy (Bruker Tensor 27, Billerica, MA, USA).

#### 4.7.1. XRD

The structure type of all the samples was checked by powder X-ray diffraction on a PANalytical X’Pert Pro diffractometer (PANalitycal, Almelo, The Netherlands) using Cu Ka radiation filtered by Ni and an X’Celerator detector. The equipment was operated at 45 kV and 30 mA and the patterns were recorded in the range 20–80° 2θ, with a step size of 0.05° and 1500 seconds per step and compared with the ICDD PDF-2 database.

#### 4.7.2. TEM

Samples were prepared by drop drying a diluted colloidal solution of NPs in ethanol onto 200 mesh Formvar-coated copper grids. Samples were observed using a Hitachi 8100 (Hitachi High-Technologies Corporation), 200 kV, LaB6 filament analytical transmission electron microscope.

#### 4.7.3. Mossbauer Spectroscopy

^57^Fe Mossbauer spectroscopy was performed at room temperature on a Wissel constant acceleration transmission mode spectrometer (Wissenschaftliche Elektronik (WissEl) GmbH, Starnberg, Germany), using a proportional counter as detector and a ^57^Co/Rh source. The resulting spectra were calibrated with an α-Fe foil and WMOSS software [40] was used for the quantitative evaluation of the spectral parameters (least-squares fitting to Lorentzian peaks). All isomeric shifts are reported relative to the centroid of the α-Fe spectrum.

#### 4.7.4. FTIR

Infrared spectra were recorded in the wave numbers range 4000–400 cm^−1^, in a Bruker Tensor 27 Fourier-transform infrared (FT-IR) spectrophotometer (Bruker Tensor 27, Billerica, MA, USA), using KBr wafers.

### 4.8. Impact of the Nanoparticles on Bacterial Adherence

Catheter pieces treated with functionalized magnetite and propolis were filled with 100 μL of an overnight bacterial culture MSSA ATCC 6538, MRSA 2, MRSA 15 and MRSA 16 (Table 3). The bacterial strains were cultivated in Brain Heart Infusion (BHI). For solid media, agar (VWR) was added at 1.5%, *w*/*v*. Bacteria were maintained in BHI with 25% (*v*/*v*) glycerol at −80 °C and, when necessary were recovered in BHI. Prior to use, bacteria were transferred to fresh BHI agar plates and incubated at 37 °C.

The previously obtained suspension of the nanomaterial was used to fabricate a modified surface in a prosthetic device. This was achieved by submerging the catheter pieces into the fluid in order to create a coating film. The catheter pieces were then dried at room temperature. This process of submerging and drying was repeated for five times of 10 s each, and the catheter pieces were sterilized by ultraviolet radiation for 20 min.

Catheter pieces treated with functionalized magnetite were filled with 100 μL of an overnight bacterial culture and left to adhere for 30 min at room temperature in a flow cabinet (Faster BH-EN 2005, Milan, Italy). Following the catheter pieces were transferred to Falcon tubes with 10 mL of Brain Heart Infusion (BHI) (Oxoid). Each tube was sonicated in a bath sonicator (J.P.SELECTA, Barcelona, Spain) for 5 min, and the catheter was immediately removed. The bacterial suspension was serially diluted using phosphate buffer saline (PBS) and the viability was determined according to the Miles and Misra technique [41]. Catheters treated with non-functionalized magnetite and catheters not treated with magnetite were used as control [12].

### 4.9. Statistical Analysis

For the statistical interpretation GraphPad Prism statistical software was used, version 5.03 (GraphPad Software, La Jolla, CA, USA). The average of six independent experiments were analyzed and compared with one-way ANOVA using Tukey’s Multiple Comparison Test of each group, followed by two-way ANOVA test for revealing significant differences among the analyzed group. Significant differences were indicated by *p* values less than 0.05.

## 5. Conclusions

The capacity of functionalized MNPs for preventing the adherence of *S. aureus* to catheters was dependent on two factors: the bacterial strain and the mode of production of the NPs. The MSSA ATCC 6538 strain was less affected by the presence of the functionalized MNPs, independently on the type of production, in contrast to the MRSA strains. For MRSA strains, the MNPs produced using Fe^3+^ and Na_2_SO_3_ solution and functionalized with oleic acid and propolis extract was most effective in the prevention of adherence of almost all MRSA strains on catheters. The hybrid MNPs produced by a hydrothermal method also had an impact in the impaired of the adherence of the strain MRSA16 on catheters. The anti-MRSA activities reported for phenolic esters (benzyl caffeate) and flavonoids (pinocembrin, galangin and their derivatives), constituents of several types of propolis and also present in our sample can be the reason, along with MNPs, for the observed activities. However, the main compound, isocupressic acid, may also have a role in the anti-adherence activities detected, although to the best of our knowledge no reference has been found upon its anti-MRSA potential. In addition, for MRSA16, the impairment of its adherence on catheters may only be attributed to the hybrid MNPs with oleic acid, since very small amount, if any at all, of propolis compounds were added to the MNPs, according to the FTIR spectra.

Nanotechnology may provide a new approach to prevent or disrupt the formation of biofilms on medical devices. The association of natural products, such as propolis, with nanotechnology may constitute an alternative to combat the formation of those biofilm in catheters by MRSA strains.

## Figures and Tables

**Table 1:** Utilization of propolis extracts as anti-Methicillin Resistant Strains of *Staphylococcus aureus* (anti-MRSA) agents.

Sample	Sample Extraction/Compounds	Strains	MIC * (mg/mL); MBC ** (mg/mL)	Major Compounds	Ref.
Twenty-four batches of propolis collected over two years (2010 and 2011) from different places in France	1. Methanol (MeOH)	Six human pathogenic bacterial strains collected by the Laboratory of Bacteriology at the University Hospital, Center of Angers, France)	0.090–>0.100; -	Pinobanksin-3-acetate, pinocembrin, chrysin, galangin, prenyl caffeate	[17]
	2. Dichloromethane (DCM)	The same reported above	0.057–0.097; -	The same reported above	
	3. Mixture of DCM, MeOH, H_2_O (31/19/4)	The same reported above	0.030; > 0.100	The same reported above	
	Pinobanksin-3-acetate	MRSA (0706C0025) MRSA (0702E0196)	>0.100; - >0.100; -		
	Pinocembrin	MRSA (0706C0025) MRSA (0702E0196)	>0.100; - >0.100; -		
	Chrysin	MRSA (0706C0025) MRSA (0702E0196)	>0.100; - >0.100; -		
	Galangin	MRSA (0706C0025) MRSA (0702E0196)	>0.100; - >0.100; -		
	Prenyl caffeate	MRSA (0706C0025) MRSA (0702E0196)	0.070; - 0.070; -		
Propolis, collected at Moravia, Czech Republic	Special propolis extract GH2002 (see the reference for the extraction method)	Ten strains	0.13–0.25; 0.5–1	-	[18]
Propolis samples from an apiary in Kamianna near Nowy Sącz in Southern Poland	Hydro-alcoholic (70%) extract of propolis	Five strains from blood clinical origin MRSA ATCC 43300	0.39–0.78; 0.78–3.13; 0.78; 3.13	Pinocembrin, kaempferol, galangin, chrysin, apigenin, quercetin, gallic acid, ferullic acid, caffeic acid, caffeic acid phenethyl ester, *p*-coumaric acid and cinnamic acid	[10]
The crude propolis and their respective ethanol extracts were sourced from the city of União da Vitória, -State of Paraná, Brazil, provided by Novo Mel^®^	Ethanol extracts	Strains (clinical isolate) were obtained from the Bacterial Library of the Microbiology Laboratory, Department of Pathology, Santa Casa de São Paulo, -School of Medical Sciences	1.42	3-[4-Hydroxy-3-(oxobutyl)-phenylacrylic acid; 3-prenyl-3(*E*)-(4-hydroxy-3-methyl-2-butenol)-5-prenylcinnamic acid; 3-prenyl-4-(2-methylpropionyloxi)cinnamic acid; 3-prenyl-4-dihydrocynamoiloxi-cinnamic acid; dihydrokaemferide; 3-prenyl-4-hydroxycinnamic acid, caffeic acid; caffeoylquinic acid 1; caffeoylquinic acid 2; caffeoylquinic acid 3; caffeoylquinic acid 4; caffeoylquinic acid 5; cinnamic acid; *p*-coumaric acid; kaempferide; kaempferol; betuletol; 2,2-dimethyl-6-carboxyethenyl-2*H*-1-benzopirane; 2,2-dimethyl-8-prenyl-2*H*-1-benzopirano-6-propenoic acid; (*E*)-3-{4-hydroxy-3-[(*E*)-4-(2,3)-dihydrocynamoiloxi-3-methyl-2-butenyl]-5-renylphenyl-2-propenoic acid; 3,4-dihydroxy-5-prenyl-cinnamic acid; 3,5-diprenyl-4-hydroxy-cinnamic acid	[19]
Two Jordanian propolis samples from two locations: University of Jordan (Type I), and Al-Hashmeah (Type II)	Type I crude aqueous methanol extracts Type II crude aqueous methanol extracts Pinobanksin-3-*O*-acetate Pinocembrin	MRSA isolated from hospitalized patients at the Jordan University	4.69	-	[20]
	Type II crude aqueous methanol extracts	The same reported above	18.75		
	Pinobanksin-3-*O*-acetate Pinocembrin		0.25; 0.25		
The propolis was from Guadalcanal Province (The Solomon Islands) for BeeVital & Herbal Apothecary (Withby, UK) ‘Pacific propolis’	Propolis extracted with 95% ethyl alcohol (EEP). A portion of EEP suspended in water/ethanol (10/1) was partitioned between *n*-hexane (HEX), ethyl acetate (EA), *n*-BuOH (BUT) and water (WAT). EA (1-16) fractions obtained from EA fractionated by gel fi ltration using Sephadex^®^ LH-20-100	One hundred and twenty clinical MRSA isolates were collected from the clinical laboratories of the New Royal Infirmary (Edinburgh, UK)	EEP: 0.064–0.128; - HEX: 0.512; - EA: 0.064–0.128 BUT: 0.128–0.256 WAT: >0.512 EA 9–EA 15: 0.016–0.064	Prenylflavanones: propolin H, propolin G, propolin D, propolin C	[21]
Purchased as ethanolic extract	Ethanolic extract of propolis (P8904, EEP, pH 7.3, Sigma, St. Louis, MO, USA)	MRSA (ATCC 33591)	1.024; - Propolis plus mupirocin for treating nares of the rabbits infected by MRSA resulted in more profound reduction in bacterial cell count and inflammatory response compared with the rest of the treatment modalities without this conjugation	-	[22]
Three samples of propolis were obtained from Croatia: sample 5587 (Zagreb) and samples 5582 and 5581 (Imotski)	Hydro-alcoholic extract (80%) of propolis (EEP), Galangin	Ten strains of MRSA	Sample 5587: 1.06; 2.00 Sample 5582: 4.98; 9.37 Sample 5581: 1.19; 2.37 Galangin: 0.16; 0.27	Flavones, flavonols, flavanones, galangin	[23]

* MIC: Minimal Inhibitory Concentration; ** MBC: Minimum Bactericidal Concentration; -: not referred by the authors.

**Table 2:** Chemical composition of hydro-alcoholic extract of Moroccan propolis.

Aromatic Acids	%	Phenolic Acid Esters	%	Flavonoids	%	Diterpenes	%	Sugars and Sugar Derivatives	%	Fatty Acids	%
Benzoic acid	0.4	Pentenyl *p*-coumarate	0.7	Pinostrobin chalcone	2.7	Ferruginol	1.2	Monosaccharides	0.4	Hexadecanoic acid	-
Hidroxybenzoic acid	0.1	Isopentenyl caffeate	1.8	Pinocembrin chalcone	5.9	Communic acid	2.7	Disaccharides	-	Octadecanoic acid	1.0
Cinnamic acid	0.3	Pentenyl caffeate	0.9	Pinocembrin	7.4	Totarol	1.1	Glycerol	0.1	Octadecenoic acid	0.5
*p*-Coumaric acid	0.3	Dimethylallyl caffeate	1.2	Pinobanksin	3.6	Imbricataloic acid	3.2	Inositol	Tr	Tetracosanoic acid	-
Dimethoxycinnamic acid	0.6	Pentenyl ferulate	0.9	Pinobanksin 3-*O*-acetate	3.4	13-*epi*-Cupressic acid	2.2	Total	0.5	Total	1.5
Ferulic acid	0.4	Benzyl ferulate	1.7	Galangin	5.3	Ferruginolon	1.2				
Isoferulic acid	0.4	Benzyl *p-*coumarate	1.3	Chrysin	3.6	Dehydroabietic acid	Tr				
Caffeic acid	0.8	Benzyl caffeate	4.7	Total	31.9	Isocupressic acid	8.1				
Total	3.3	Caffeic acid phenetyl ester	1.7			Junicedric acid	1.8				
		Cinnamyl ferulate	0.4			Total	21.5				
		Cinnamyl caffeate	1.2								
		Total	16.5								
Standard deviation does not succeed 6% for any of the constituents											

**Table 3:** *Staphylococcus aureus* strains.

Bacteria	Origin	Source
*Staphylococcus aureus* ATCC 6538 (MSSA ATCC 6538)	Wound	American Type Culture Collection
*Staphylococcus aureus* methicillin-resistant 2 (MRSA 2)	Clinical	UAlg, CBMR. Portugal
*Staphylococcus aureus* methicillin-resistant 15 (MRSA 15)	Clinical	UAlg, CBMR. Portugal
*Staphylococcus aureus* methicillin-resistant 16 (MRSA 16)	Clinical	UAlg, CBMR. Portugal
